# The Institutional Worker

**Published:** 1911-11-18

**Authors:** 


THE
### INSTITUTIONAL WORKER
**Being a Special Supplement to "The Hospital"**

### Editor's Notices.

**Contributions:**

Contributions should be written, or preferably typed, on one side of the paper only, and all articles sent in are accepted upon the distinct understanding that they are forwarded to The Hospital only.

The Editor cannot undertake to return MSS. not used, but when a stamped directed envelope is enclosed the MSS. may be returned if a special request is made.

Accepted articles and paragraphs of news will be paid for after publication at the scale rate.

**Address:**

To prevent delay all contributions and letters on editorial business must be addressed exclusively to the Editor, "The Hospital," 29 Southampton Street, Strand, London, W.C. It is important that this regulation shall be strictly observed.

**Correspondence:**

Correspondence on all subjects is invited. The name and address of correspondents must be given as a guarantee of good faith, but not necessarily for publication.

**Special Articles:**

Special articles are invited, and questions, inquiries, and paragraphs upon all matters relating to the work, administration, and management of general, special, mental, fever, cottage and convalescent hospitals, sanatoria, homes, institutions, societies and organisations for the treatment or care of the sick, injured and dependents of all classes. Every matter affecting the interests and welfare of the staff of all grades working in these institutions will receive special consideration.

**Administrative Medicine, Research and Items of News:**

Special terms will be paid for approved articles on subjects in this wide field by experts who have made some section of it their special study and interest. We wish to give prominence to every movement tending to advance accuracy of diagnosis, efficiency in treatment, and the development of modern methods to eradicate disease and promote the welfare of the suffering.

**A Bureau of Information:**

We invite inquiries and applications for information and help in providing for that numerous class of sufferers who require special care or house-room which they cannot obtain in their own homes or secure for themselves. **We cannot, however, prescribe or recommend practitioners.**

**Books for Review:**

Publishers are particularly requested to send advance proofs of any new books of importance whenever possible, as the Editor has made arrangements to publish immediate reviews on a new plan.

**Photographs, Plans, Blocks, and Illustrations:**

It is requested that wherever possible MSS. may be accompanied by illustrations in any of the above forms. The name of the sender and of the article to which it belongs should be written on the back of each photograph, drawing, or block for purposes of identification.

**Local Papers:**

Newspapers containing reports or news paragraphs should be marked and addressed to the Sub-Editor.

**The Coupon System:**

A coupon will be found at the bottom of the third inside page of the cover each week. This coupon must be attached to every question or inquiry to which an answer is desired in The Hospital.

### Manager's Notices.

The Hospital, with its new features and interesting articles, has been received with abundant signs of appreciation. One of our subscribers writes from Cardiff as follows: "The value of The Hospital to me as a voluntary worker is considerable, and it cannot be estimated at the price of the publication." Several commendatory letters have reached this office conveying congratulations on the excellence of The Hospital from the readers' point of view, and we hope to make the paper indispensable to every institutional worker throughout the country.

So great has been the demand for The Hospital during the last few weeks that it has been found necessary to give instructions to the printers to increase the number. While we regret that some of our readers have been unable to purchase a copy owing to the paper being sold out at the bookstalls, we have now made arrangements for an increased supply, and there should no longer be any difficulty in obtaining The Hospital every Friday.

**Subscriptions,** which may begin at any time, are payable in advance. Cheques and Post-Office orders, crossed London County and Westminster Bank, Covent Garden branch, should be made payable to the Manager of The Hospital and addressed to him at The Hospital Building, 28 and 29 Southampton Street, Strand, London, W.C.

**Advertisements:**

All advertisements for The Hospital must reach the Manager not later than Wednesday morning in each week. For scale of charges see pages v and 2.

**Classified Advertisements:**

All small advertisements will be classified, for this section of the paper is widely read and of special interest. We wish to encourage those wanting appointments who are attracted to institutional work, and therefore offer them the reduced rate of *twenty-one words and under for* 1s., and *for every ten and part of ten words beyond*, 6*d*.

### POINTS FOR INSTITUTION AUTHORITIES.

The economical management of an Institution depends very greatly upon purchasing supplies in the cheapest market.

It is often impossible locally to secure the highest quality at the lowest prices.

In consequence, a section will be reserved in this part of the paper for announcements of Institutions inviting Tenders for Contracts.

This will enable Authorities of Institutions to secure tenders for supplies from all over the country, and ensure purchases at the lowest possible prices consistent with quality.

The charge for Tenders is 6s. per inch, and for this small outlay many pounds may be saved on supplies of all descriptions.

**Special Rates** will be quoted for those institutions which agree to send all their vacancy, contract, and public notices to The Hospital each year, so as to make it a file of such announcements for ready reference.

### POINTS FOR TRADE ADVERTISERS.

The Hospital is the organ of Institutions, whether of a Voluntary Character or those under Local Government, Poor Law and Municipal Authorities.

It is read by all Executive Officials and those responsible for the Construction, Management, and Equipment of Public Establishments.

It is also the Journal of the worker engaged in the Medical, Health and Preventive services.

There is no medium more appropriate and effective in the promotion of business in these directions than The Hospital.

### APPOINTMENTS VACANT.

### Poor-Law Vacancies.

### Appointments Vacant.

Assistant Laundry Superintendent (£23 18s.), St. George-in-the-East Guardians. Mr. R. M. Lochner, C. to G., Raine Street, Old Gravel Lane, E. Nov. 28.

Assistant Night Nurse for Children's Homes (£22), Bermondsey Guardians. Mr. E. Pitts Fenton, C. to G., 283 Tooley Street, S.E. Nov. 20.

Assistant Nurse (£22), Ormskirk Union. A. Dickinson, C. to G., Ormskirk. Nov. 23.

Assistant Nursery Attendant (£14), Barnet Union. The Matron of the Workhouse, Barnet. Nov. 20.

Assistant Nurse (£20), Stafford Isolation Hospital. The Town Clerk, Stafford.

Assistant Nurse (£25), Louth Union. Mr. F. C. Chard, C. to G., Louth. Nov. 18.

Assistant Nurse (£25), Guildford Union. W. S. V. Cullerne, C. to G., Commercial Road, Guildford. Nov. 24.

Assistant Nurse (£28), Eastry Union. F. S. Cloke, C. to G., Sandwich. Nov. 20.

Assistant Nurse (£31), West Ham Union. T. Smith, C. to G., Union Road, Leytonstone, N.E. Dec. 6.

Charge Attendant on Male Lunatics (£50), Kingston Union. Mr. C. W. Dash, C. to G., Kingston-on-Thames.

Charge Nurse (£32), Middlesbrough Union. J. J. Dales, C. to G., Poor Law Offices, Middlesbrough. Nov. 22.

Charge Nurse (£24), City of London Union. Matron of the Infirmary, 42 Clifden Road, Median Road, N.E.

Charge Nurse (£30), South Shields Union. H. W. Coulson, C. to G., Union Offices, Barrington Street, S. Shields. Nov. 22.

Charge Nurse (£28), Brighton Guardians. B. Burfield, C. to G., Parochial Offices, Brighton. Nov. 28.

Charge Nurse (£30), West Ham Union. T. Smith, C. to G., Union Road, Leytonstone, N.E. Dec. 6.

Class Sister (£50), West Ham Union. T. Smith, C. to G., Union Road, Leytonstone, N.E. Dec. 6.

Female Attendant (£20), Islington Guardians. Matron, Islington Workhouse, St. John's Road, Upper Holloway.

Female Attendant (£24), Billericay Union. Mr. C. E. Lewis, C. to G., Brentwood. Nov. 20.

Foster-Mother (£25), York Union. Mr. G. Sykes, C. to G., York. Nov. 21.

Infirm Attendant (£25), Wigan Union. Mr. H. G. Ackerley, C. to G., Wigan. Nov. 21.

Infants' Helper (£16), Hackney Union. Matron of the Branch Workhouse, Brentwood.

Junior Clerk (£50), Southwark Union. Mr. S. Wood, C. to G., Ufford Street, Blackfriars Road, S.E. Nov. 23.

Male Attendant (£30), York Union. Mr. G. Sykes, C. to G., York. Nov. 21.

Male Imbecile Attendant (£25), Leicester Guardians. Mr. H. Mansfield, C. to G., Leicester. Nov. 21.

Male Lunatic Attendant (£30), Holborn Union. Master of the Workhouse, 1a Sheperdess Walk, N.

Male Nurse (£35), Bermondsey Guardians. E. Pitts Fenton, C. to G., 283 Tooley Street, S.E. Nov. 20.

Master and Matron (£30 each), Gower Union. Mr. H. J. Ind, C. to G., Fisher Street, Swansea. Nov. 27.

Master's Clerk (£25), Hammersmith Guardians. The C. to G., 206 Goldhawk Road, Shepherd's Bush, W. Nov. 27.

Matron's Assistant (£35), West Derby Union. Mr. H. P. Cleaver, C. to G., Brougham Terrace, Liverpool. Nov. 21.

MIDWIFERY NURSE (£30), Hackney Union. F. R. Coles, C. to G., Clerk's Office, Sidney Road, Homerton, N.E. Nov. 29.
NURSE (£28), West Ashford Union. Mr. J. M. Poncia, C. to G., Ashford, Kent. Nov. 18.
NURSE (£25), Hartley Wintney Union. Mr. W. H. Wright, C. to G., Odiham, Hants.
NURSE (£35), Sleaford Union. Mr. E. Clements, C. to G., Sleaford.
NURSES' ASSISTANT (£20), Redruth Union. Mr. T. Peter, C. to G., Redruth. Nov. 21.
NURSERY AND YARD ATTENDANT (£26), Basford Union. Mr. H. Stone, C. to G., Basford, Notts.
NURSERY ATTENDANT (£22), Bermondsey Guardians. Mr. E. Pitts Fenton, C. to G., 283 Tooley Street, S.E. Nov. 20.
PROBATIONER (£12), Mitford and Launditch Union. Matron of the Workhouse, Gressenhall, East Dereham.
PROBATIONERS (£10), Wakefield Guardians. H. Beaumont, C. to G., 47 Kirkgate, Wakefield. Nov. 25.
PROBATIONER NURSE (£10), Burton-on-Trent Union. C. F. Chamberlin, C. to G., Union Offices, Burton-on-Trent. Nov. 20.
PROBATIONER NURSES, Kingston-upon-Hull Guardians. R. H. Winter, C. to G., St. Mary's Chambers, Hull. Nov. 18.
PROBATIONER (£5), Mansfield Union. W. S. Cockerham, C. to G., 105 Stockwell Gate, Mansfield. Nov. 23.
RELIEVING OFFICER AND COLLECTOR (£120 and commission), Burnley Union. Mr. J. S. Horn, C. to G., Burnley. Nov. 22.
SCHOOLMISTRESS (£70), Brighton Guardians. Mr. B. Burfield, C. to G., Brighton. Nov. 18.
SECOND ASSISTANT NURSE (£20), Walsingham Union. Mr. R. S. Butcher, C. to G., Fakenham. Nov. 21.
SISTER (£35), Wandsworth Union. Mr. F. W. Piper, C. to G., St. John's Hill, Wandsworth, S.W.
STAFF NURSE (£24), Hackney Union. Matron of the Workhouse, Sidney Road, Homerton, N.E.
STAFF NURSES (£31), West Ham Union. Mr. T. Smith, C. to G., Leytonstone, N.E. Nov. 23.
SUPERINTENDENTS OF VAGRANT WARDS (married couple) (£50 and £20), Billericay Union. Mr C. E. Lewis, C. to G., Brentwood. Nov. 20.
TAILOR AND INDUSTRIAL TRAINER (£40), Portsmouth Guardians. Mr. E. H. Mitchell, C. to G., Portsmouth. Nov. 21.
THIRD ASSISTANT BOOKKEEPER (£30), South Manchester Guardians. Mr. D. S. Bloomfield, C. to G., All Saints, Manchester.
WARD SISTER (£30), Hammersmith Guardians. The C. to G., 206 Goldhawk Road, Shepherd's Bush, W.

### Situations Vacant.

ASSISTANT COOK (£25). Camberwell Guardians. Mr. C. S. Stevens, C. to G., Peckham Road, S.E. Nov. 20.
ASSISTANT LAUNDRESS (£20), Greenwich Union. Mr. T. Cutter, C. to G., East Greenwich.
ASSISTANT LAUNDRESS (£30), Kingston Union. Mr. C. W. Dash, C. to G., Kingston-on-Thames.
ASSISTANT MALE COOK (£30), Fulham Guardians. Mr. E. J. Mott, C. to G., 129 Fulham Palace Road, W. Nov. 20.
COOK AND GENERAL ASSISTANT (£25), Lymington Union. Mr. J. D. Rawlins, C. to G., Lymington.
COOK (£27), Burton-on-Trent Union. Mr. C. F. Chamberlin, C. to G., Burton-on-Trent. Nov. 20.
FEMALE COOK (£27 10s.), Preston Union. Mr. J. Clarke, C. to G., Preston. Nov. 23.
FEMALE COOK (£25), Wangford Union. Mr. S. W. Rix, C. to G., Beccles. Nov. 20.
FEMALE COOK FOR OFFICERS (£23), Farnham Union. Mr. E. Crundwell, C. to G., Farnham. Nov. 22.
LAUNDRESS (15s. weekly), Basford Union. Mr. H. Stone, C. to G., Basford, Notts.
LAUNDRESS (£25), Camberwell Guardians. Mr. C. S. Stevens, Peckham Road, S.E. Nov. 20.
LAUNDRESS (£30), King's Norton Union. Mr. R. J. Curtis, C. to G., Guildhall Buildings, Birmingham.
MASTER AND MATRON, PORTERS, MALE ATTENDANTS, COOKS, MASTER'S CLERK, STOREKEEPER, BAKER, ENGINEER, STOKERS, LAUNDRY SUPERINTENDENT, ETC., City of London Union. Mr. E. R. Woodward, C. to G., Bartholomew Close, E.C.
PORTRESS AND LAUNDRY SUPERINTENDENT (£20), Runcorn Union. Mr. G. F. Ashton, C. to G., Runcorn.
RELIEF MAID (£20), Bermondsey Guardians. Matron of the Infirmary, Lower Road, Rotherhithe.
STOKER, ETC. (£36), Romford Union. Mr. C. Bloomfield, C. to G., Romford. Nov. 20.

### Institutional and Administrative Vacancies.

### Appointments Vacant.

MATRON (£40), Llangollen Cottage Hospital. Hon. Secretary. Nov. 25.
MATRON (£45), Exmouth Cottage Hospital. Hon. Secretary. Nov. 20.
NURSE MATRON (£40), Victoria Cottage Hospital, Wimborne. Hon. Secretary. Nov. 22.
OPHTHALMIC SISTER (£30), King Edward VII.'s Hospital, Cardiff. Matron. Nov. 18.
STAFF NURSE (£28), Colwyn Bay Cottage Hospital. Hon. Secretary. Nov. 22.
THEATRE SISTER (£35), London Homœopathic Hospital. Apply Matron.

### Municipal Vacancies.

### Appointments Vacant.

ASSISTANT NURSE (£20), Borough of Stafford Isolation Hospital. Apply Town Clerk, Stafford.
ASSISTANT CLERK (£52), Essex and Colchester Asylum. Mr. H. H. Gepp, Clerk to the Visitors, Brentwood.
BUILDING INSPECTOR (£2 weekly), Colne T.C. Mr. A. Varley, Town Clerk, Colne. Nov. 20.
CLERK OF WORKS (Sewerage) (£3 3s. weekly), Scunthorpe and Crosby Joint Sewerage Board. Mr. G. E. Davy, Clerk to the Board, High Street, Scunthorpe. Nov. 21.
CLERK OF WORKS (Schools) (£3 3s. weekly), Leicester T.C. Mr. T. Groves, Secretary for Education, Leicester. Nov. 30.
DISTRICT SURVEYOR (£150), North Riding C.C. Mr. W. G. Bryning, County Surveyor, Northallerton. Nov. 30.
FINANCIAL CLERK (£100), Isle of Wight R.D.C. Mr. H. E. Stratton, Clerk to the Council, Newport, Isle of Wight. Nov. 20.
GAS AND WATER MANAGER (£180), Atherton U.D.C. Mr. W. Garnett, Clerk to the Council, Atherton. Nov. 22.
HEALTH VISITOR (£100), Metropolitan Borough of Poplar. L. Potts, Town Clerk, High Street, Poplar. Dec. 4.
HEALTH VISITOR (£75), Stoke-on-Trent Corporation. E. B. Sharpley, Town Clerk, Stoke-on-Trent. Nov. 23.
HEALTH VISITOR (£100), Finsbury B.C. Mr. G. W. Preston, Town Clerk, Rosebery Avenue, E.C. Nov. 20.
HIGHWAYS SURVEYOR (£130), Isle of Wight R.D.C. Mr. H. E. Stratton, Clerk to the Council, Newport, Isle of Wight. Nov. 20.
LADY PROBATIONER (£18), Croydon Mental Hospital, Medical Superintendent of the Hospital, Upper Warlingham, Surrey.
SURVEYOR (£120), Halesowen R.D.C. Mr. E. H. Grove, Clerk to the Council, Great Carnbow, Halesowen. Nov. 20.

### Situations Vacant.

CARETAKERS (married couple) (£2 weekly joint), Houghton-le-Spring R.D.C. Isolation Hospital. Mr. R. B. Marley, Clerk to the Council, Houghton-le-Spring. Nov. 21.
CARETAKERS FOR ISOLATION HOSPITAL, Atherstone R.D.C. Mr. W. A. Hatton, Clerk to the Council, Atherstone.
COOK (£28-30), Coventry City Hospital. Apply Matron. Dec. 6.

